# An Intriguing Case of Acute Encephalopathy: Lesson Learned from a Constipated Man

**DOI:** 10.7759/cureus.6678

**Published:** 2020-01-16

**Authors:** Munish Sharma, Humayun Anjum, Chinthaka P Bulathsinghala, Palla Rivi De Silva, Salim Surani

**Affiliations:** 1 Internal Medicine, Corpus Christi Medical Center, Corpus Christi, USA; 2 Pulmonary/Critical Care, Corpus Christi Medical Center, Corpus Christi, USA; 3 Pulmonary Critical Care, Corpus Christi Medical Center, Corpus Christi, USA; 4 Internal Medicine, Texas A&M Health Science Center, Bryan, USA

**Keywords:** acute encephalopathy, non-cirrhotic hyperammonemia, constipation, lactulose

## Abstract

Hyperammonemia is a common cause of encephalopathy encountered in an intensive care unit (ICU). Absence of pre-existing liver disease may misguide a clinician and cases of non-cirrhotic hyperammonemia may be missed in ICU leading to life-threatening outcomes such as cerebral edema and herniation. A critical care physician must look beyond liver cirrhosis as a cause of hyperammonemia so that infrequent but potentially reversible causes of encephalopathy are not missed, and patient treatment is not jeopardized.

## Introduction

Acute encephalopathy refers to global cerebral dysfunction in the absence of underlying primary structural disease of the brain [1]. It is a common cause of admission to an intensive care unit (ICU). Most of the causes of acute encephalopathy are potentially reversible but delayed or missed diagnosis of underlying etiology can prolong hospital stay, increase mortality and adversely affect functional and cognitive function in the long run [2]. Thus, it is imperative for a clinician to promptly recognize and treat any potentially reversible cause of acute encephalopathy in a patient admitted to ICU.

## Case presentation

**Figure 1:**
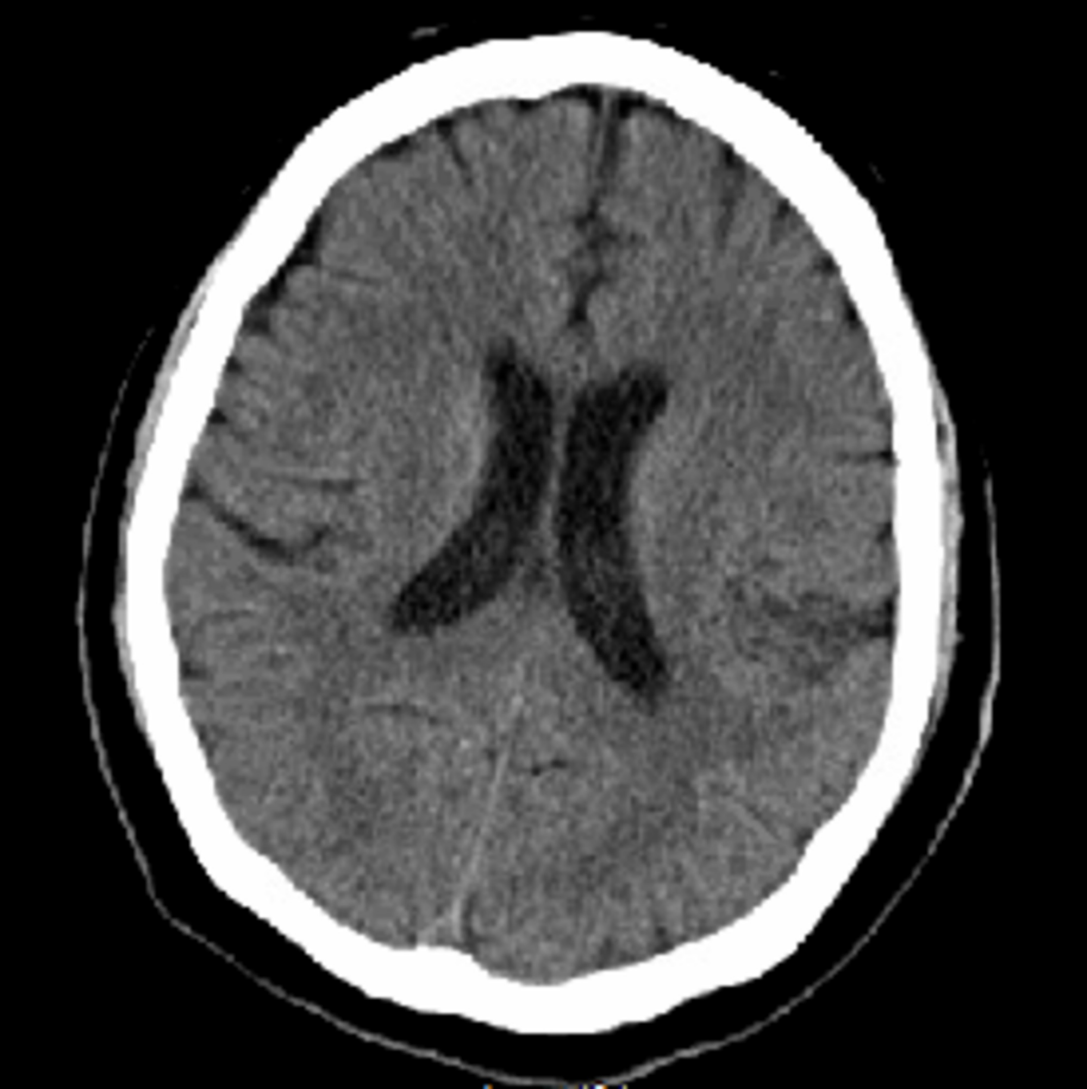
Computed tomography of the head without contrast did not reveal acute intracranial pathology.

An 82-year-old man with the past medical history of congestive heart failure (ejection fraction 30-35%), non-ischemic cardiomyopathy and history of a permanent pacemaker for sick sinus syndrome and normal functional and mental status at baseline was brought to our hospital for acute onset of lethargy and confusion for two days. On examination, his blood pressure was 150/77 mmHg, heart rate 64 beats/minute, respiratory rate 16/minute, temperature 36.9 degree Celsius, saturation 100% on room air. His Glasgow Coma Score was 9. He had no signs of meningeal irritation; no focal motor deficit was noted, no facial droop, no tremor of extremities, normal flexor plantar response with normal deep tendon reflexes. We were unable to completely assess the sensory system and all 12 cranial nerves due to encephalopathy. His cardiovascular, respiratory and abdominal examination did not reveal any abnormalities. There were no skin rashes and no significant lymphadenopathy. The family denied any head trauma, fever, chills, sick contacts, and recent travel. No new medications were prescribed to the patient before this presentation. His total leucocyte count was 6.26 x 10^3^/µL, blood urea nitrogen 8 mg/dl (reference range: 6-20), serum creatinine 0.82 mg/dl (reference range: 0.6-1.00), serum sodium 138 mmol/L (reference range: 133-145), serum potassium 3.8 mmol/L (reference range: 3.5-5.5), magnesium 2.1 mg/dl (reference range: 1.7-2.2), phosphorus 4.1 mg/dl (reference range: 2.5-4.5), serum glucose 134 mg/dl (reference range: 70-130), aspartate aminotransferase 20 units/L (reference range: 15-37), alanine aminotransferase 31 units/L (reference range: 30-65), serum lactic acid 1.8 mmol/L (reference range: 0.5-2.2). His vitamin B12 and serum folic acid level were normal. Urinalysis and urine culture ruled out urinary tract infection. His computed tomography (CT) of the head without contrast did not reveal any acute intracranial pathology (Figure 1).

**Figure 2:**
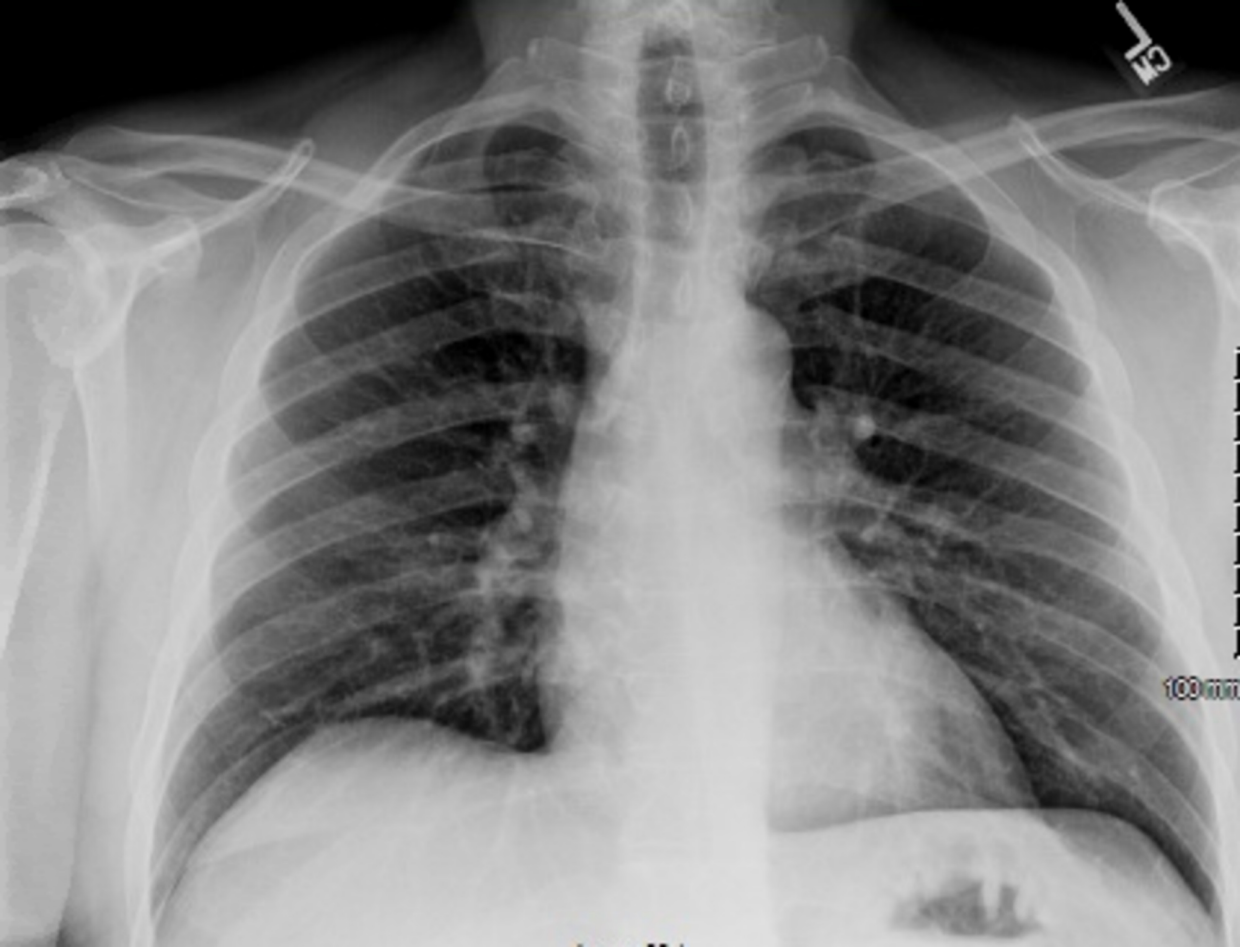
Chest X-ray did not show any acute abnormality.

A urine drug screen was non-revealing; serum alcohol level was 3 mg/dL (reference: 0-10). Electrocardiogram showed normal sinus rhythm without any ST-T wave changes and chest X-ray did not show any evidence of acute radiographic abnormality (Figure 2).

Arterial blood gas showed ph 7.44, pCO2 35.2 mmHg, pO2 98 mmHg, bicarbonate 23.2 mmol/L on room air. Blood cultures did not show any growth. We did not perform a lumbar puncture, as there was no suspicion for acute meningoencephalitis based on history and examination. Electroencephalogram showed mild to moderate slowing with theta frequency predominantly consistent with mild to moderate encephalopathy.

**Figure 3:**
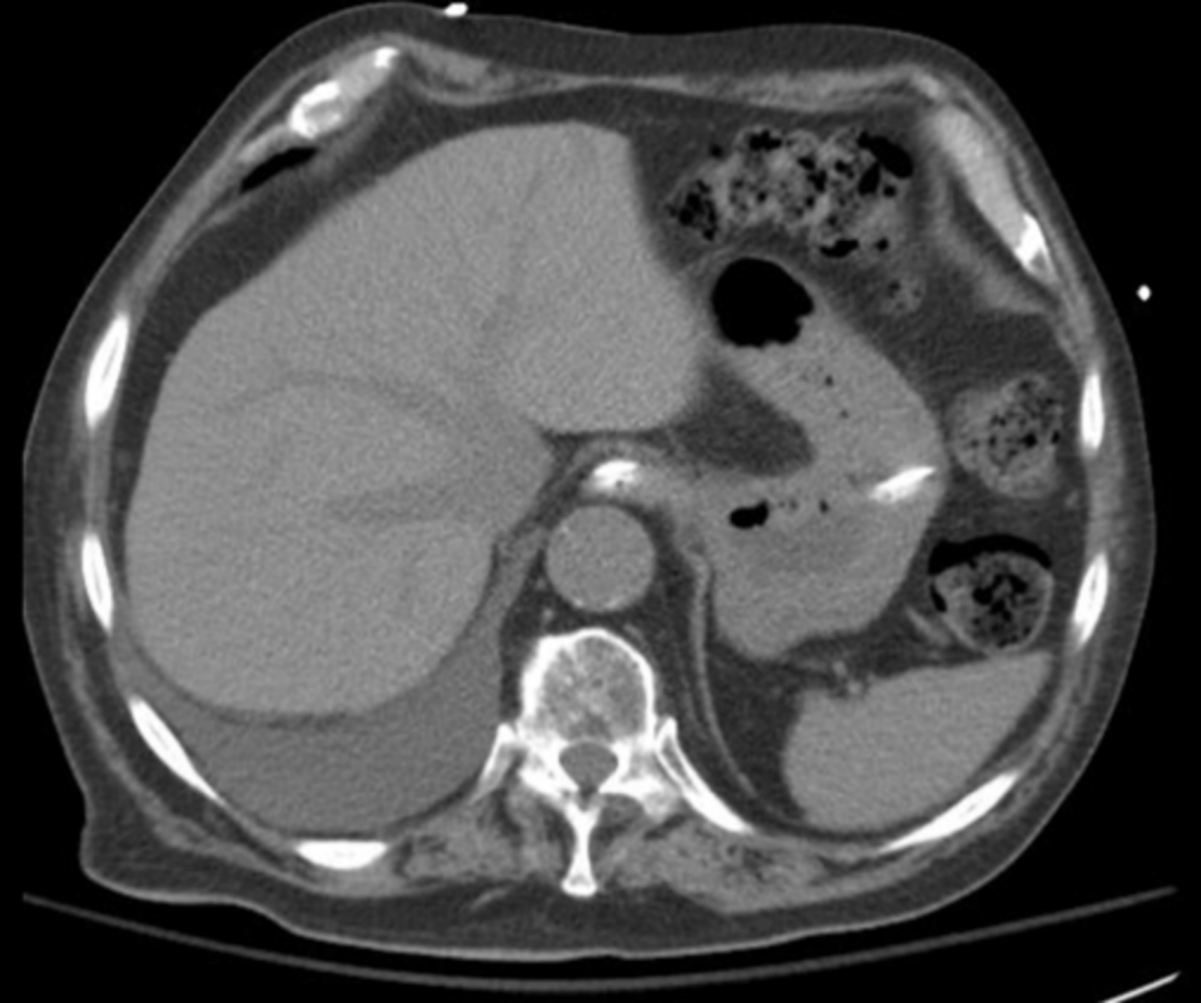
Computed tomography of abdomen showing normal liver echotexture.

His serum ammonia level was elevated to 274 μmol/L (reference range: 11-35). CT of the abdomen without contrast did not reveal cirrhotic changes of the liver (Figure 3).

**Figure 4:**
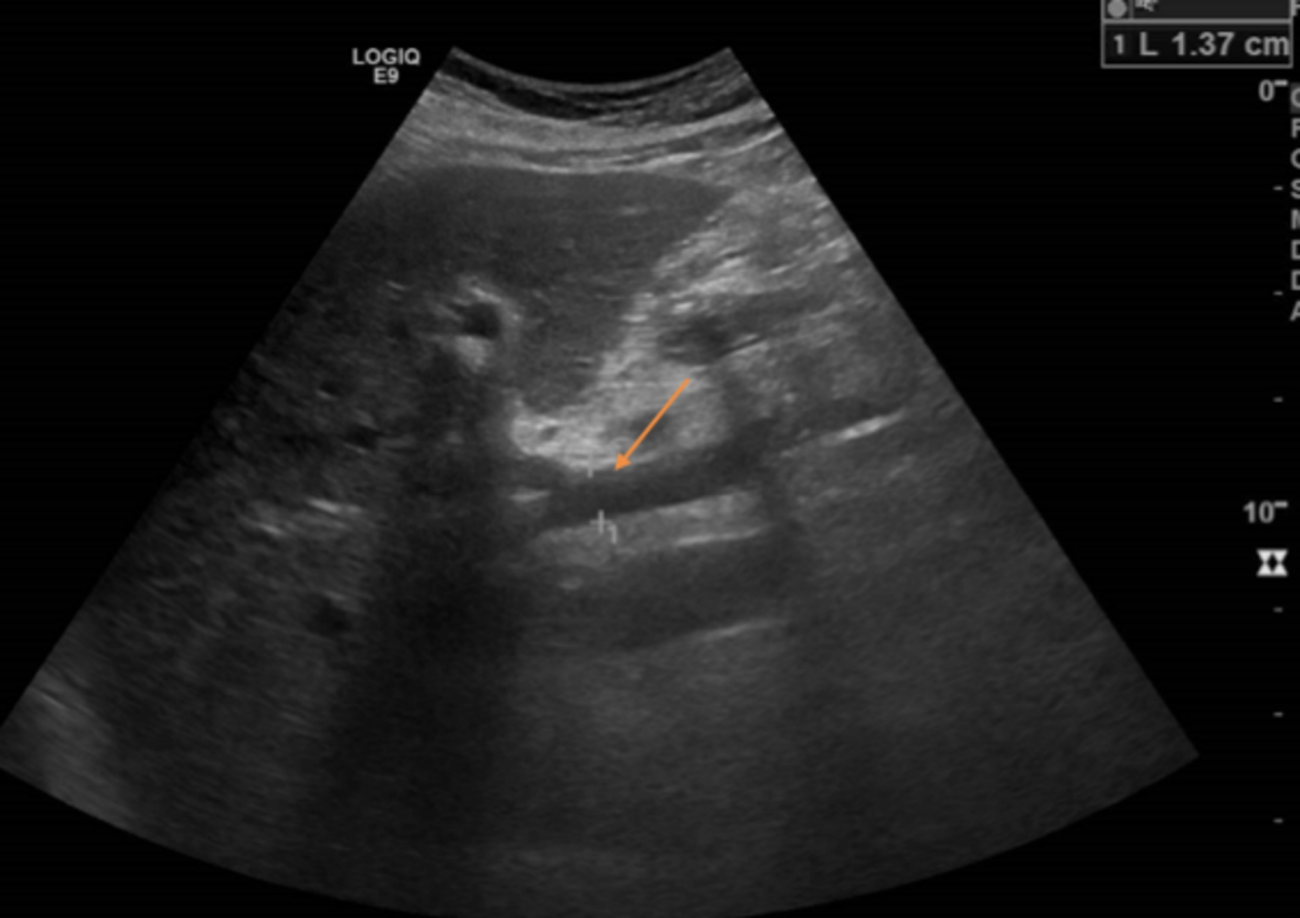
Portal vein measuring 1.37 cm (red arrow).

A liver ultrasound with Doppler showed the normal size and echo texture of the liver and mildly dilated main portal vein. It also showed patent hepatic and portal veins with normal directional flow seen through the portal vein (Figure 4).

Hepatitis A IgM antibody, hepatitis B surface antigen, hepatitis B core IgM antibody were non-reactive while hepatitis C antibody titer was less than 0.1 signal to cut off ratio (reference range <0.8). Esophagogastroduodenoscopy did not reveal esophageal varices or any source of active bleeding. Serum and urine amino acid concentration obtained by liquid chromatography-mass spectrometry did not reveal aminoacidopathy. On careful review of the patient’s history before hospital admission, it was revealed that he was severely constipated for at least a week before the onset of encephalopathy. The cause of severe constipation could not be exactly determined. There was no new medication prescribed or use of sodium valproate at home. We treated the patient with lactulose through nasogastric tube 20 gm every six hours to maintain 3-4 loose stools per day. The patient’s serum ammonia level trended down to 46 and thereafter to 30 μmol/L after four days. Mental status improved significantly with a decrease in hyperammonemia and relief of constipation. He was subsequently transferred out of ICU and strongly recommended to avoid constipation with as-needed use of lactulose.

## Discussion

Most of the cases of hospital admission due to acute encephalopathy are metabolic in origin. Hyperammonaemia is an extremely commonly encountered cause of acute encephalopathy. Ninety percent of hyperammonemia-induced encephalopathy is associated with underlying liver disease [3]. In the remaining 10% of cases, non-cirrhotic causes of hyperammonemia should be determined and appropriately treated. Ammonia causes encephalopathy due to its toxic effect on neurons. It increases neutral amino acid uptake by the brain through the enhancement of L-amino acid transporter at the blood-brain barrier. As a result of increased concentration of neutral amino acids such as tryptophan, phenylalanine, and tyrosine, synthesis of neurotransmitters like serotonin, dopamine, and norepinephrine is decreased [4,5]. Hyperammonemia can also cause brain edema due to increased intracellular osmolarity resulting from the increased conversion of ammonia to glutamine [6]. Some studies in animal models have also described oxidative stress due to elevated ammonia resulting in cerebral edema [7]. Ammonia inhibits the generation of inhibitory and excitatory postsynaptic potentials and alters neural electric activity [8].

Non-cirrhotic hyperammonemia can be either from increased ammonia production or decreased ammonia elimination. Infections by urea producing bacteria such as Proteus mirabilis, Klebsiella species, Morganella morganii can cause hyperammonemia [9]. Multiple myelomas, bone marrow transplantation, acute leukemias, and chemotherapy and organ transplantation can cause hyperammonemia [10]. Catabolic states induced by seizures, extreme exercise, trauma, steroid use, and gastrointestinal bleed can also increase ammonia production [11]. Ureterosigmoidostomy, late-onset inborn errors of metabolism, intra and extra hepatic portosystemic shunts and drugs such as valproic acid, ribavirin, carbamazepine, and salicylate can also cause hyperammonemia [12-14].

In our patient, there was no established identifiable cause of hyperammonemia discussed above. He had severe constipation for over a week before the onset of encephalopathy. His encephalopathy was likely due to increased absorption of ammonia into the mesenteric blood supply due to very slow transit constipation. Such absorption of ammonia most likely overwhelmed hepatic excretion. A similar case scenario and hypothesis has been previously described [15]. We primarily used lactulose, a nonabsorbable disaccharide, to treat hyperammonemia and the patient improved significantly after maintaining three to four bowel movements daily. Lactulose helps decrease endogenous ammonia production, reduces intestinal pH, entraps ammonia as non-diffusible ammonium in the gut lumen and increases fecal nitrogen load by the direct cathartic effect. If ammonia had persistently elevated to >100 mol/L, we could have used conventional or peritoneal dialysis to enhance ammonia elimination [16].

## Conclusions

Absence of pre-existing liver disease may misguide a clinician and cases of non-cirrhotic hyperammonemia may be missed leading to life-threatening outcomes such as cerebral edema and herniation. Persistent unresolved encephalopathy increases hospital stay, increases morbidity and mortality and leads to significant cognitive and functional decline in the long run. Thus, clinicians must routinely check serum ammonia in patients with acute encephalopathy of unclear etiology and must perform workup to determine potentially treatable causes.
